# Time Trend Analysis of Early Term Births in Greece (1980-2023): Persistent High Rates Raise Public Health Concerns

**DOI:** 10.7759/cureus.80606

**Published:** 2025-03-15

**Authors:** Nikolaos Vlachadis, Chryssi Christodoulaki, Nikolaos Machairiotis, Dimos Sioutis, Ioannis Tsakiridis, Themistoklis Dagklis, Konstantinos Louis, Georgios Petrakos, Maria Siori, Periklis Panagopoulos, Dimitrios Panagiotopoulos

**Affiliations:** 1 Department of Obstetrics and Gynecology, General Hospital of Messinia, Kalamata, GRC; 2 Third Department of Obstetrics and Gynecology, National and Kapodistrian University of Athens, Medical School, Attiko Hospital, Athens, GRC; 3 Third Department of Obstetrics and Gynecology, Aristotle University of Thessaloniki, Medical School, Hippokrateio Hospital, Thessaloniki, GRC; 4 Health Center of Vyronas, National Health System of Greece, Athens, GRC; 5 Department of Obstetrics and Gynecology, General Hospital of Messinia, Kyparissia, GRC

**Keywords:** births, duration of gestation, early term, early term births, full term, greece, time trends

## Abstract

Introduction: Neonates born at 37-38 weeks of gestation have been shown to face a relatively higher risk of mortality and various morbidities compared to those born at full term (39-41 weeks). The aim of this study was to conduct a comprehensive analysis of early term birth rate (ETBR) trends in Greece from 1980 to 2023.

Materials and methods: Data on live births in Greece from 1980 to 2023 were obtained from the Hellenic Statistical Authority (ELSTAT), based on birth certificate records. A total of 4,595,020 live births were categorized by gestational age. The annual ETBR was calculated as the number of live births occurring at 37-38 completed gestational weeks (from 37+0 to 38+6 weeks) per 100 total live births. Additionally, ETBR was calculated separately for 37 and 38 gestational weeks, as well as the overall birth rate < 39 weeks of gestation. Time trends were evaluated using joinpoint regression analysis.

Results: The ETBR decreased with an annual percent change (APC) of -1.4 (95% confidence interval (CI): -1.8 to -0.9, p = 0.004) between 1980 and 1994, followed by a sharper decline with an APC of -12.8 (95% CI: -14.4 to -8.5, p = 0.005) during 1994-1997. Subsequently, the ETBR increased with an APC of 3.2 (95% CI: 2.0 to 5.6, p = 0.016) from 1997 to 2004. This trend reversed again from 2004 to 2010, with an APC of -2.6 (95% CI: -5.9 to -1.1, p = 0.016), while in the most recent period (2010-2023), the ETBR stabilized (p = 0.630), fluctuating between 41.9% in 2010 and 44.3% in 2012 and 2013. During the study period (1980-2023), the ETBR at 37 weeks increased by an average of 7.1% per year, whereas the ETBR at 38 weeks decreased by an average of -2.1% annually. The overall birth rate < 39 weeks of gestation has remained consistently above 50% over the past two decades (2004-2023). In 2023, the ETBR was 43.7%, while a total of 56.0% of neonates were born < 39 gestational weeks.

Conclusions: This study provides a comprehensive analysis of ETBR in Greece, revealing persistently high levels that exceed those of all developed countries. The elevated rate is likely driven by factors such as maternal demographics and obstetrical practices. The alarmingly high levels of ETBR, combined with the country's extremely high preterm birth rates, result in a substantial burden of neonatal morbidity and an increased risk of potential chronic diseases. These findings underscore an enormous public health challenge.

## Introduction

The duration of pregnancy plays a critical role in shaping both the immediate and long-term health outcomes for newborns. Preterm birth, defined as delivery before 37 weeks of gestation, has long been acknowledged as a significant risk factor for various adverse effects. However, emerging research suggests that the outcomes of pregnancy exist on a spectrum rather than a binary classification. This perspective has led to the recognition of early term birth, which refers to live births that occur between 37 weeks (37+0) and 38 weeks and six days (38+6) of gestation [1-3].

Epidemiological studies show that while early term newborns have a notably lower risk of complications when compared to preterm infants, they still encounter higher rates of adverse outcomes than those born at full term (39 weeks or later). Despite the relatively lower mortality and morbidity rates associated with early term births, the public health implications of these deliveries are significant due to their much higher incidence compared to preterm births. This underscores the importance of targeted interventions aimed at reducing unnecessary early term deliveries and promoting optimal gestational timing [1,4].

Growing evidence indicates that infants born at 37-38 weeks of gestation have increased neonatal morbidity compared to those born at full term after 39 weeks. These early-term infants are more likely to require admission to neonatal intensive care and experience respiratory complications. Additionally, the implications extend to long-term health issues, including neurocognitive and developmental challenges that can persist into adulthood [1,5,6].

Epidemiological data indicate a correlation between high rates of preterm and early term births in developed countries, attributed to shared underlying causes and risk factors, including pregnancy complications, maternal demographics, and medical practices that lead to provider-initiated deliveries without rigorous medical justification [1]. Notably, Greece has experienced a troubling increase in preterm birth rate (PBR) over recent decades, surpassing levels observed in other high-income nations [7]. The aim of this study was to conduct a comprehensive analysis of early term birth rate (ETBR) trends in Greece from 1980 to 2023, identifying temporal patterns and evaluating the current public health challenges. 

## Materials and methods

Study population

The study included all live births occurring at ≥ 20 completed weeks of gestation in Greece between 1980 and 2023, categorized by gestational age. Data were obtained from the Hellenic Statistical Authority (ELSTAT) [8], derived from the analysis of national birth certificate records. These records are considered the most comprehensive and reliable source of birth data in Greece, as they are legally mandated to be completed for every live birth in the country and are systematically collected and validated by ELSTAT.

Inclusion and exclusion criteria

A total of 4,605,769 live births were recorded in Greece during the study period (1980-2023). Of these, 4,595,020 live births (99.77%) with documented gestational age were included in the analysis. The remaining 10,749 births (0.23%) were excluded due to missing or incomplete gestational age information.

Sample size

The final sample size consisted of 4,595,020 live births with available gestational age data, representing 99.77% of all recorded live births during the study period. This large sample size would provide robust statistical power for detecting trends and changes over time, as well as ensuring the precision of the rates examined.

Study parameters

The primary outcome measure was the annual ETBR, defined as the number of live births occurring at 37-38 completed gestational weeks (from 37+0 to 38+6 weeks) per 100 total live births. To provide a more granular analysis, the ETBR was further stratified into births at 37 completed weeks and births at 38 completed weeks, each calculated per 100 live births. Additionally, the overall birth rate at < 39 weeks of gestation was calculated per 100 live births to assess the total burden of vulnerable newborns, including both preterm (< 37 weeks) and early term (37-38 weeks) births, in the Greek population. These measures were chosen to align with international standards for perinatal health reporting and to facilitate comparisons with other studies.

Statistical analysis

Data were analyzed using Microsoft Excel, version 2010 (Microsoft Corp., Redmond, Washington, United States) for initial data organization and descriptive statistics. Trend analysis was conducted using the Joinpoint Regression Program, version 5.2.0 (National Cancer Institute, Bethesda, Maryland, United States). This software employs a log-linear model to identify joinpoints, which represent specific years marking statistically significant shifts in trends. The analysis estimated the annual percent change (APC) for each segment between two joinpoints. The maximum number of allowed time segments was set at seven, based on the study duration and the need to balance model complexity with interpretability. Additionally, the average annual percent change (AAPC) for the entire study period (1980-2023) was calculated as a weighted average of the APCs derived from the joinpoint model, with weights corresponding to the length of each APC interval. Results were reported with 95% confidence interval (CI), and statistical significance was set at a p-value < 0.05. Sensitivity analyses were conducted to confirm the robustness of the findings, including testing alternative joinpoint configurations and selecting the best-fitting model for the data.

Ethical considerations

The study utilized publicly available, anonymized, aggregated data from national birth records, and no individual-level data were accessed, ensuring compliance with ethical standards for the use of secondary data.

## Results

**Figure 1:**
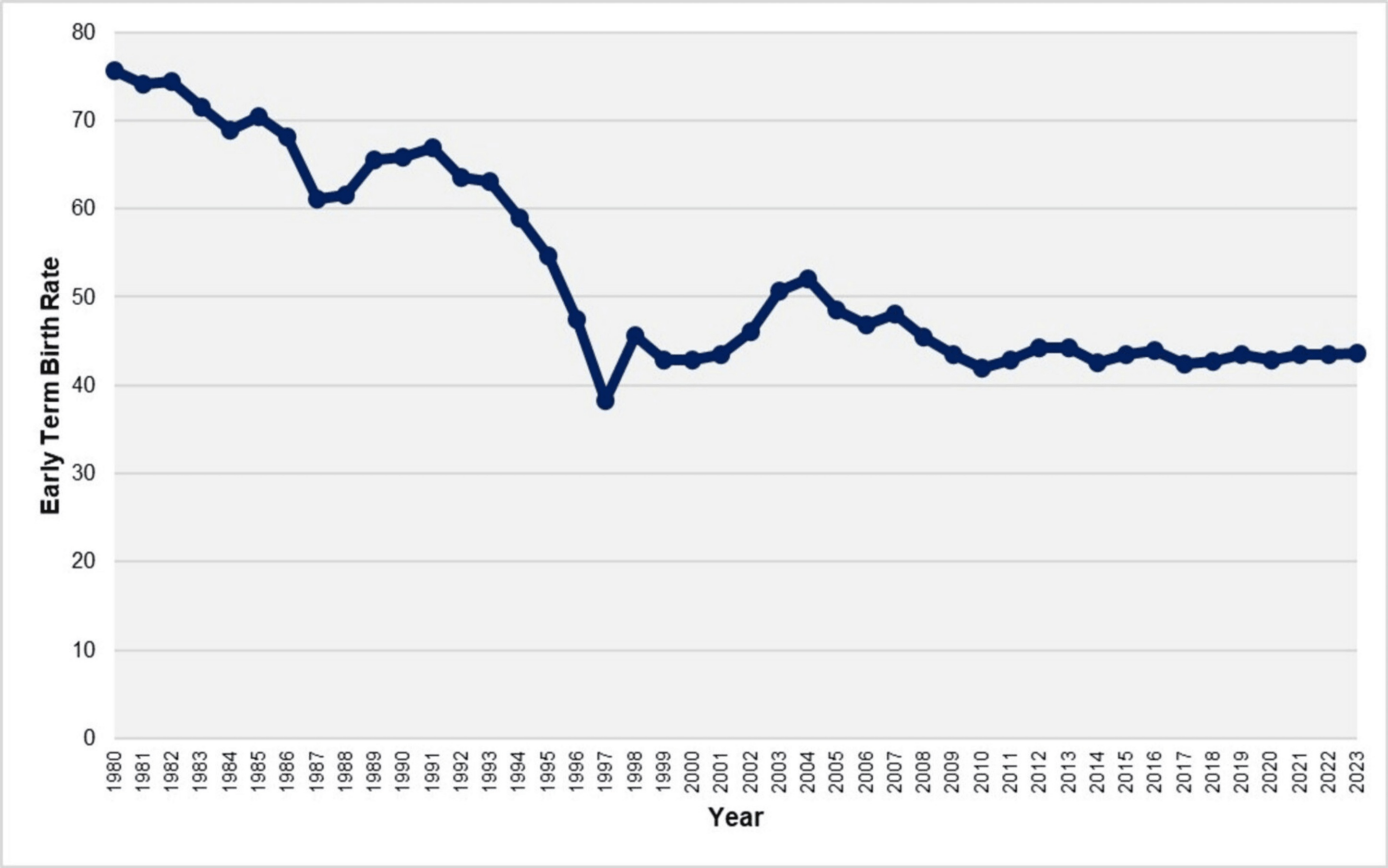
Early term birth rates (per 100 live births) in Greece, 1980-2023.

**Figure 2:**
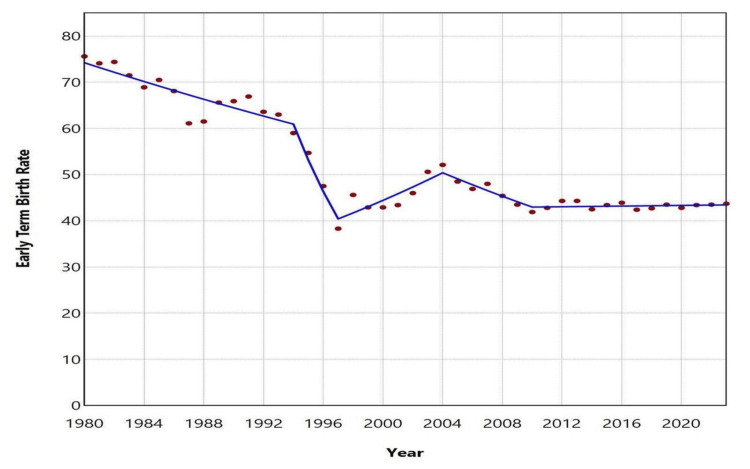
Trends in early term birth rates in Greece, 1980-2023

From 1980 to 2023, among 4,595,020 births with registered gestational age, there were 2,467,294 early term births, resulting in an overall ETBR of 53.7 per 100 live births. The ETBR decreased substantially from its peak of 75.6% in 1980 to a low of 38.3% in 1997, marking a 49% reduction. This decline occurred in two phases: from 1980 to 1994, the rate decreased at an APC of -1.4 (95% CI: -1.8 to -0.9, p = 0.004), followed by a sharper decline from 1994 to 1997 with an APC of -12.8 (95% CI: -14.4 to -8.5, p = 0.005). Subsequently, the rate increased by 36%, reaching 52.1% in 2004, with an APC of 3.2 (95% CI: 2.0 to 5.6, p = 0.016) during the 1997-2004 period. However, the trend reversed again from 2004 to 2010, with a decline of 20% to 41.9% in 2010, reflecting an APC of -2.6 (95% CI: -5.9 to -1.1, p = 0.016). In the most recent period, from 2010 to 2023, the ETBR stabilized (APC = 0.1, 95% CI: -0.4 to 1.3, p = 0.630), fluctuating between 41.9% in 2010 and 44.3% in 2012 and 2013 (Figures 1-2, Table 1).

**Table 1:** Trends in early term birth rates in Greece, 1980-2023

Segment	Annual percent change	95 confidence interval	P-value
1980-1994	-1.4	-1.8 to -0.9	0.004
1994-1997	-12.8	-14.4 to -8.5	0.005
1997-2004	3.2	2.0 to 5.6	0.016
2004-2010	-2.6	-5.9 to -1.1	0.016
2010-2023	0.1	-0.4 to 1.3	0.630

**Figure 3:**
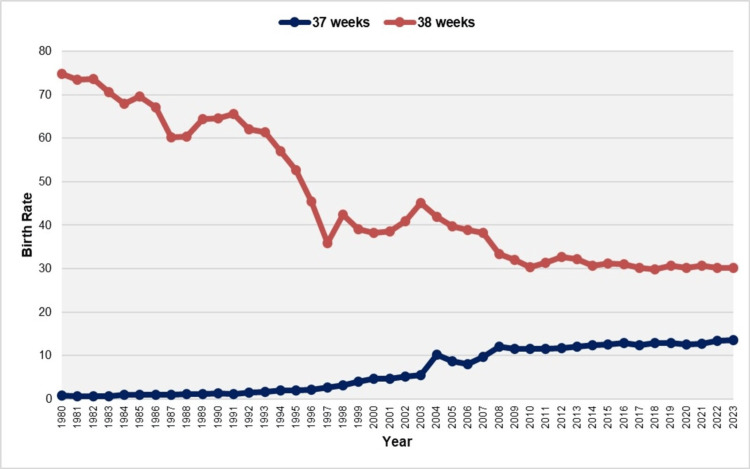
Early term birth rates at 37 and 38 weeks (per 100 live births) in Greece, 1980-2023

**Figure 4:**
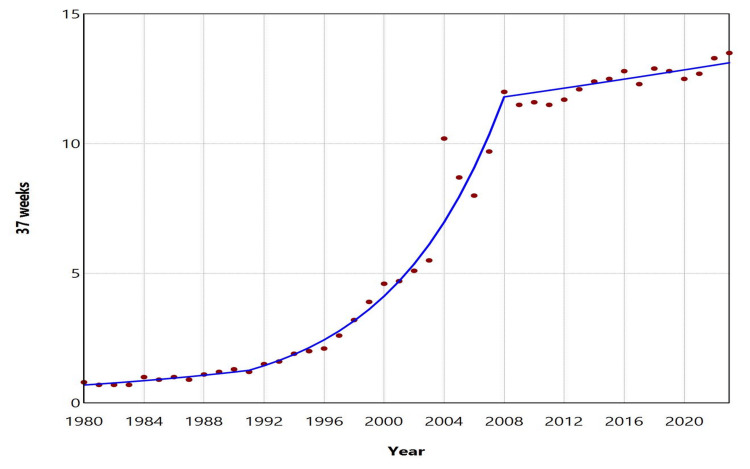
Trends in early term birth rates at 37 weeks in Greece, 1980-2023

**Figure 5:**
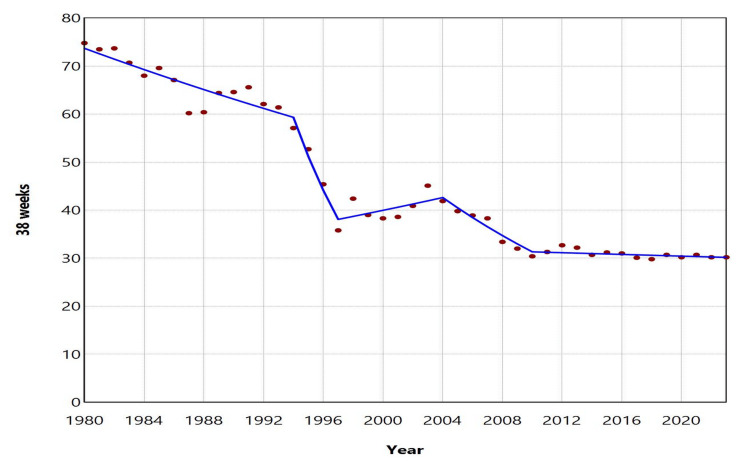
Trends in early term birth rates at 38 weeks in Greece, 1980-2023

The ETBR at 37 weeks increased with an AAPC of 7.1 (95% CI: 6.7 to 7.4, p < 0.001) from 1980 to 2023. This rising trend was observed with an APC of 5.6 (95% CI: 3.2 to 7.4, p = 0.002) during the period from 1980 to 1991, followed by a steep increase with an APC of 14.1 (95% CI: 13.0 to 15.6, p < 0.001) from 1991 to 2008. However, between 2008 and 2023, the increase was statistically nonsignificant (APC = 0.7, 95% CI: -0.6 to 1.8, p = 0.225). In contrast, the ETBR at 38 weeks showed a declining trend over the same period, with an AAPC of -2.1 (95% CI: -2.2 to -1.9, p < 0.001). The downward trend continued from 1980 to 2010, except for the period from 1997 to 2004, while from 2010 to 2023, the rate remained statistically stable. Between 2010 and 2023, the ETBR at 37 weeks fluctuated from 11.5% (2011) to 13.5% (2023), the highest value recorded during 1980-2023, while the ETBR at 38 weeks ranged from 29.8% (2018) to 32.7% (2012). In 2023, 30.8% of all early term births occurred at 37 weeks, while 69.2% occurred at 38 weeks (Figures 3-5, Tables 2-3).

**Table 2:** Trends in early term birth rates at 37 weeks in Greece, 1980-2023

Segment	Annual percent change	95% Confidence interval	P-Value
1980-1991	5.6	3.2 to 7.4	0.002
1991-2008	14.1	13.0 to 15.6	< 0.001
2008-2023	0.7	-0.6 to 1.8	0.225

**Table 3:** Trends in early term birth rates at 38 weeks in Greece, 1980-2023

Segment	Annual percent change	95% confidence interval	P-value
1980-1994	-1.5	-1.9 to 1.0	< 0.001
1994-1997	-13.7	-15.5 to -9.6	< 0.001
1997-2004	1.6	0.4 to 4.2	0.009
2004-2010	-5.0	-8.9 to 3.3	< 0.001
2010-2023	-0.3	-0.8 to 0.5	0.385

**Figure 6:**
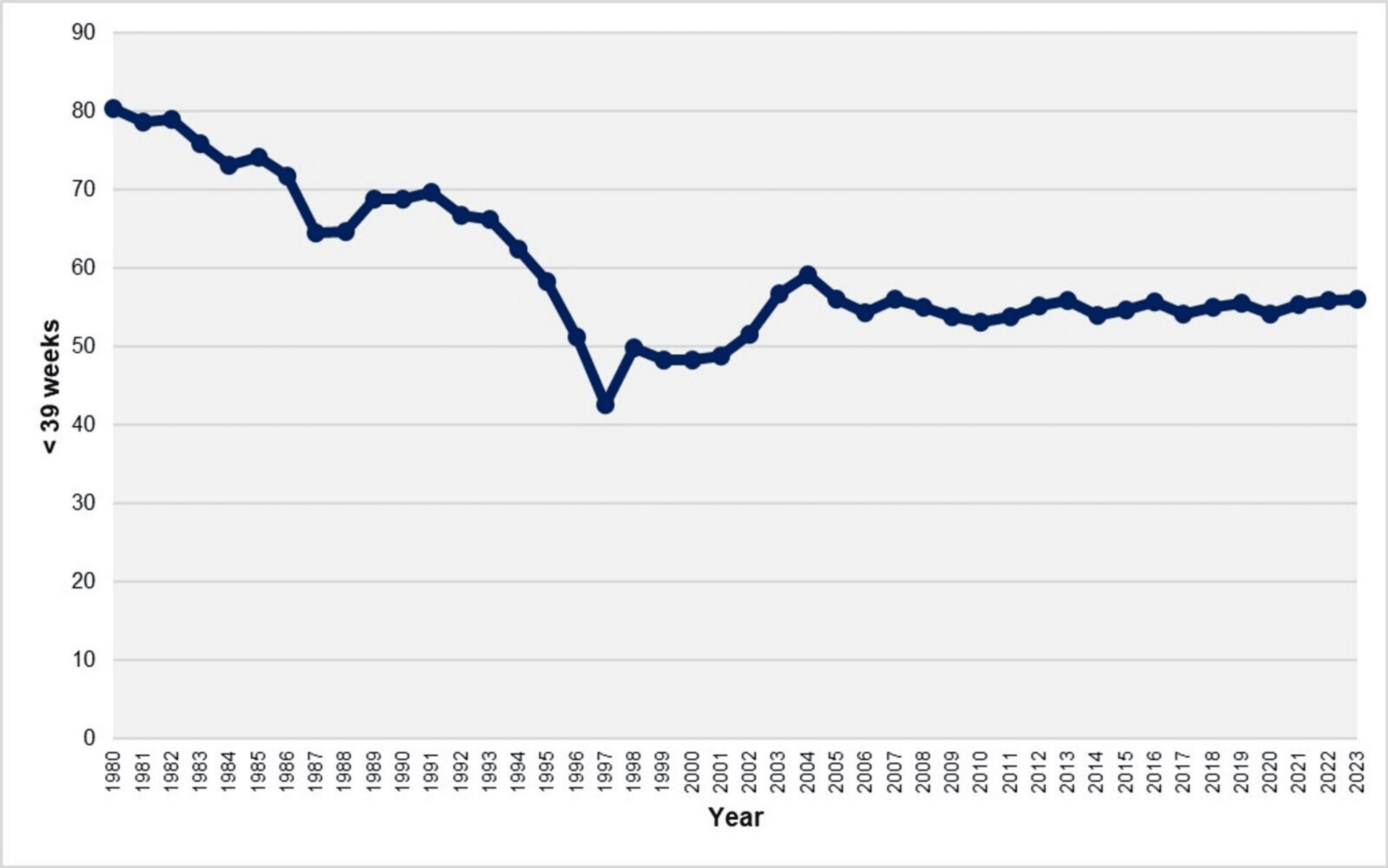
Birth rates at < 39 weeks (per 100 live births) in Greece, 1980-2023

**Figure 7:**
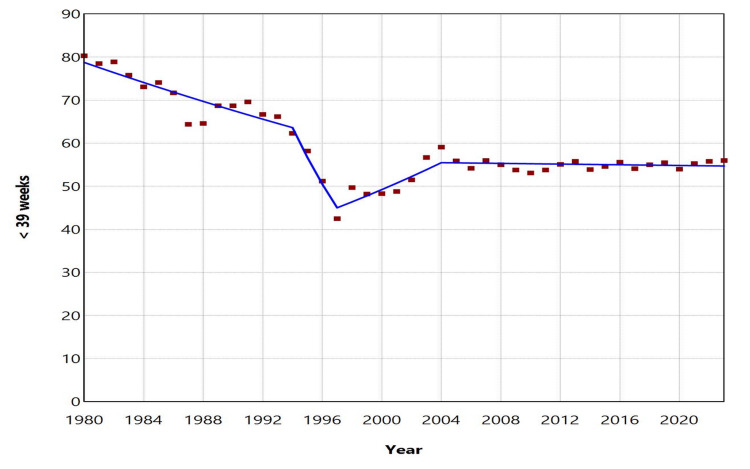
Trends in birth rates at < 39 weeks in Greece, 1980-2023

The birth rate at < 39 weeks of gestation declined significantly from 1980 to 1997, with an APC of -1.5% (95% CI: -1.9 to -1.0, p < 0.001) from 1980 to 1994, followed by a steeper decline from 1994 to 1997 (APC = -10.9, 95% CI: -12.6 to -7.2, p < 0.001). This trend reversed from 1997 to 2004, with a significant increase in the birth rate (APC = 3.0, 95% CI: 2.0 to 6.2, p < 0.001). Over the past two decades (2004-2023), the trend has remained stable, with rates consistently above 50% and ranging from 53.1% in 2010 to 59.1% in 2004. In 2023, 56.0% of live births in Greece occurred before 39 weeks of gestation, with 77.9% of these classified as early term (37-38 weeks) and 22.1% as preterm (< 37 weeks) (Figures 6-7, Table 4).

**Table 4:** Trends in birth rates at < 39 weeks in Greece, 1980-2023

Segment	Annual percent change	95% confidence interval	P-value
1980-1994	-1.5	-1.9 to -1.0	< 0.001
1994-1997	-10.9	-12.6 to -7.2	< 0.001
1997-2004	3.0	2.0 to 6.2	< 0.001
2004-2023	-0.1	-0.4 to 0.2	0.555

## Discussion

The analysis of official national data has provided insight into the long-term trends of early term births in Greece over the last four decades. Following fluctuations during the 30-year period from 1980 to 2010, the ETBR in the country has remained essentially unchanged at very high levels, exceeding 40%, from 2010 to 2023. The persistence of the ETBR at consistently elevated levels poses a central challenge for perinatal medicine in Greece and carries significant public health implications.

The results of an analysis of more than 4.5 million live births over a 44-year period revealed very high birth rates between 37+0 and 38+6 weeks of gestation in the Greek population. The ETBR experienced a downward trend from 1980 to 1997, but subsequently increased from 1997 to 2004, surpassing 50% of total births. Following this period, there was an improvement in the rate until 2010; however, since then, early term births in Greece have remained stable, fluctuating between approximately 42 and 44 per 100 live births.

Furthermore, the separate analysis of the ETBR by week of gestation revealed contrasting trends at 37 and 38 completed weeks. Specifically, the ETBR at 37 weeks exhibited a strong upward trend, averaging a 7.1% increase per year and reaching a peak of 13.5% in 2023, although it has shown stabilizing tendencies since 2008. In contrast, the ETBR at 38 weeks demonstrated an overall downward trend, resulting in 2023 seeing more than 30% of early term births occurring at 37 weeks.

**Figure 8:**
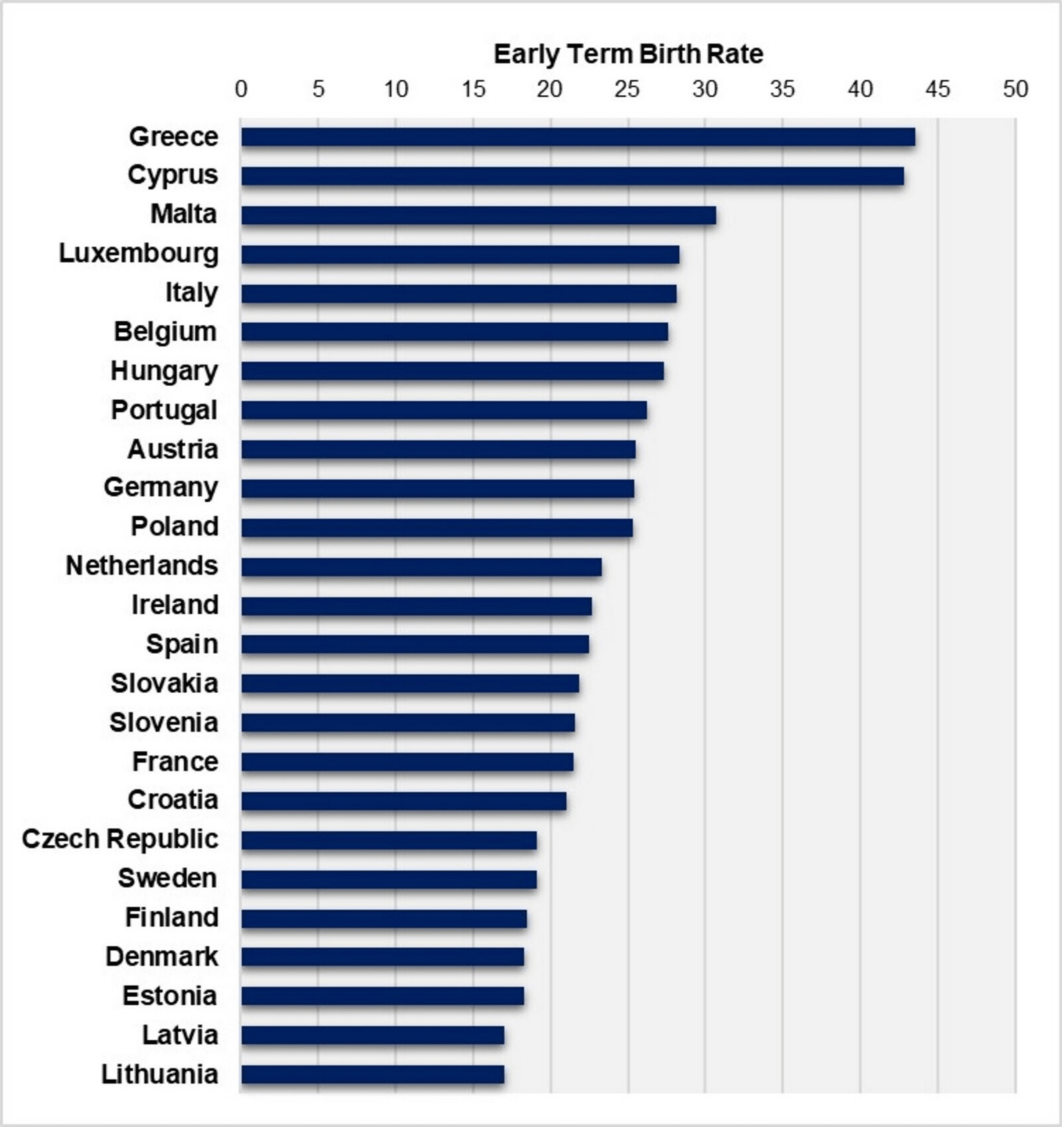
Early term birth rates (per 100 live births) in 25 European Union countries, 2019 Created by author Nikolaos Vlachadis using data from European Perinatal Health Report (EUROPERISTAT) [11] for 24 countries, with additional data for Greece from this study. Data for Bulgaria and Romania are not included.

An overview of studies with comparative data shows that Greece consistently has the highest ETBR among developed countries. In a study comparing the ETBR for singletons in the United States, Canada, and four Scandinavian countries (Denmark, Finland, Norway, Sweden) from 2006 to 2015, it was found that the ETBR was higher in North America, with an average of 26.9% in the United States and 25.3% in Canada during 2006-2014. In Northern Europe, the ETBR ranged from an average of 16.8% in Finland (2006-2015) to 18.8% in Denmark (2006-2010) [4]. In contrast, the ETBR in Greece during the same period was significantly higher, with rates ranging from approximately 42% to 49% for the total population of live births, showing no signs of improvement. Furthermore, in 2022, the percentage of all newborns delivered at 37-38 weeks of gestation was 29.3% in the United States, while in Greece, it was nearly 50% higher, at 43.5% [9]. Another study analyzing data from 34 developed countries (not including Greece) reported a median ETBR for single pregnancies in 2010 of 22.2%, ranging from 15.6% in Ireland to 30.8% in Malta, while in comparison, Greece had an ETBR of 41.9% among all live births the same year [10]. Additionally, Greece has the highest ETBR among European Union (EU) countries, as evidenced by a comparison of Greek national data with the European Perinatal Health Report (EUROPERISTAT), which did not include data from Greece. In 2019, the median ETBR in EU countries was 23.3%, while the rates for Cyprus and Greece were outliers, with values exceeding 40% (Figure 8) [11].

**Figure 9:**
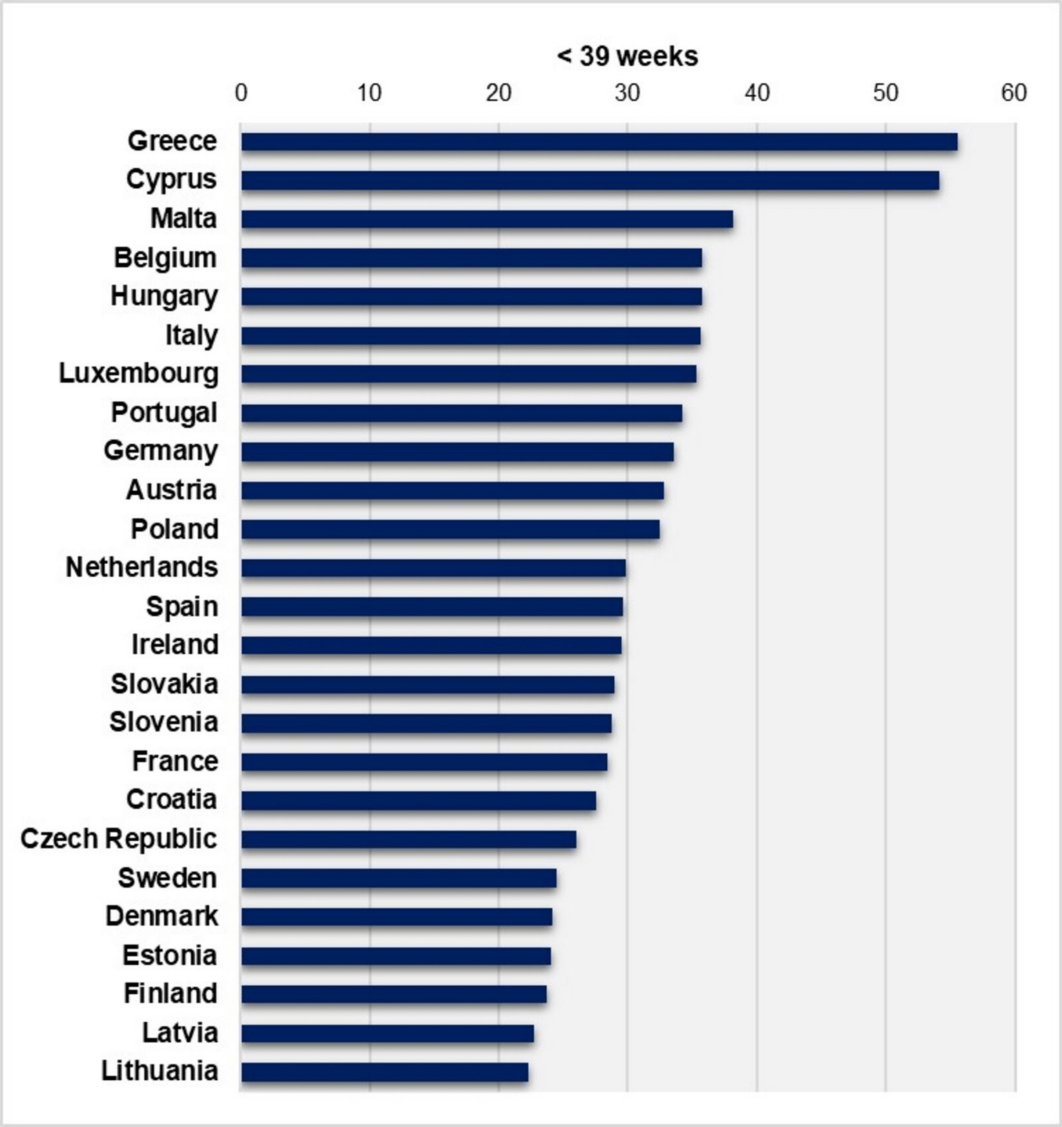
Birth rates < 39 weeks (per 100 live births) in 25 European Union countries, 2019 Created by author Nikolaos Vlachadis using data from European Perinatal Health Report (EUROPERISTAT) [11] for 24 countries, with additional data for Greece from this study. Data for Bulgaria and Romania are not included.

Additionally, this study analyzed the variation in birth rates before the completion of 39 weeks of gestation, combining both the PBR and the ETBR. This approach aimed to highlight the overall burden of vulnerable newborns within the Greek population. The total birth rate < 39 weeks of gestation, after fluctuations during the period 1980-2004, has remained unchanged over the past two decades (2004-2023), consistently at extremely high levels exceeding 50%, ranging from approximately 53% to 59%. This is an expected outcome of the combined negative impact of an extremely high PBR [7] and an exceedingly high ETBR, which together result in the majority of newborns in Greece being at risk for morbidities during infancy, childhood, and even adulthood. A comparison with EUROPERISTAT data reveals that in 2019, the birth rate < 39 weeks of gestation in Greece was the highest among European countries, similar to that of Cyprus. The median rate across Europe was 29.8%, while Greece and Cyprus were outliers, being the only countries with rates exceeding 50% (Figure 9) [11].

Demographic factors are likely to have an impact on the high ETBR in Greece. Since the 1980s, Greece has seen a steep drop in birth rates [12], accompanied by a notable shift in its fertility patterns toward advanced maternal age [13]. There is a modest positive correlation between the ETBR and advanced maternal age, particularly for women over 40 years old [14]. Countries with elevated PBR, such as Greece, also tend to have high ETBR [10]. The similar values of PBR and ETBR in Greece and Cyprus, which differ significantly from those of other developed countries, suggest a shared etiology. This likely corresponds to similar cultural and behavioral factors along with comparable obstetric practices. This could be explained by the common ethnic (Hellenic) ancestry and close ties between the two countries, as well as the fact that obstetricians in both countries largely share a similar clinical training and approach. Furthermore, in Greece, multiple births have reached epidemic levels [15]. Although a strong correlation between multiple pregnancies and the incidence of prematurity in Greece has been documented [16], their role in shaping high ETBR levels is likely negligible. The impact of multiple pregnancies on ETBR has not been studied in the literature. However, it appears that, at least in developed countries, the incidence of early term births does not differ significantly between singleton and multiple deliveries. For example, in 2022, the ETBR in the United States was 29.3% overall and 29.1% for singleton births [9,14]. Although there are no relevant published data for Greece, it can be inferred from the data in Cyprus that high ETBR is primarily due to high rates in singleton births rather than multiple births. For example, in Cyprus in 2019, the PBR for multiple pregnancies was 74.8%, meaning that the ETBR for multiple births was less than 25%, while the overall ETBR was 42.8% [11]. This suggests that the ETBR for singletons was higher than that for multiple pregnancies. The associations between ETBR, maternal age, and multiple births in Greece warrant further investigation.

Neonates born at 37-38 weeks of gestation, while typically considered full-term, have been documented to be at a relatively increased risk for mortality and morbidity compared to those born between 39-41 weeks [4,17]. This heightened incidence of adverse outcomes during the neonatal and infant periods is associated with respiratory distress, metabolic disorders, and nutritional issues [5,18]. Long-term complications can include neurodevelopmental disorders, as well as increased mortality and morbidity in adulthood, which are linked to cardiovascular, metabolic, and respiratory diseases [3,19,20]. National data in Greece persistently reveal high levels of ETBR exceeding 40%, which, when combined with preterm births, result in over 50% of newborns being classified as at risk. An additional concerning finding is the rise in the birth rate at 37 weeks, which is associated with poorer outcomes, although this trend appears to have stabilized recently. In 2023, more than one in four newborns in the country were born before completing 38 weeks of gestation.

Certain strategies, if implemented, could contribute to lowering ETBR and enhance both infant and long-term health outcomes. Addressing modifiable risk factors for early term delivery is crucial, particularly those related to obstetric practices. Strict adherence to specific criteria for early delivery before 39 weeks of gestation is essential. Guidelines recommend avoiding nonmedically indicated early-term deliveries. Furthermore, a holistic approach is necessary, encompassing epidemiological tracking of maternal and perinatal risk factors, as well as long-term monitoring of infants born before 39 weeks, extending into adulthood [21,22].

In this study, we analyzed data from over 4.5 million live births, based on records from national birth certificates, allowing for a comprehensive evaluation of early term births in Greece. This is the first study in the literature to focus specifically on the prevalence and trends of early term births in Greece, and it holds particular significance because relevant data from Greece are not included in international publications. A key limitation of this study is the challenge of accurately dating gestational age. While gestational age is typically precise in pregnancies resulting from assisted reproductive technologies or those closely monitored during early pregnancy, it may be less reliable in cases with inadequate prenatal care. These limitations could introduce misclassification bias when categorizing preterm and early term births [7].

## Conclusions

This study offers a comprehensive analysis of ETBR in Greece over a 44-year period, revealing persistently high levels that exceed those of other developed countries. Despite historical fluctuations, the ETBR in Greece has remained alarmingly high, with recent rates consistently surpassing 40%. The combined impact of extremely high PBR and ETBR results in more than 50% of all births occurring before 39 weeks of gestation, placing the majority of newborns in the country at risk for both short- and long-term health complications. These findings underscore the urgent need for coordinated efforts to address modifiable risk factors and ensure evidence-based practices to reduce early deliveries and their associated health burdens in Greece.
